# The development of an indirect ELISA for the detection of goose parvovirus antibodies using specific VP3 subunits as the coating antigen

**DOI:** 10.1186/s12917-019-2027-1

**Published:** 2019-08-01

**Authors:** Karolina Tarasiuk, Lucyna Holec-Gąsior, Bartłomiej Ferra, Andrzej Rapak

**Affiliations:** 1 Department of Poultry Diseases, National Veterinary Research Institute, Partyzantów 57 Avenue, 24-100 Puławy, Poland; 2 Department of Microbiology, Gdańsk University of Technology, Gabriela Narutowicza 11/12, 80-233 Gdańsk, Poland; 3 Laboratory of Tumor Molecular Immunobiology, Ludwik Hirszfeld Institute of Immunology and Experimental Therapy, Polish Academy of Sciences, Rudolfa Weigla 12, 53-114 Wrocław, Poland

**Keywords:** ELISA, Goose parvovirus, Recombinant VP3 protein

## Abstract

**Background:**

In Poland, the leader in goose production in Europe, goose parovirus infection, or Derzsy’s disease (DD), must be reported to the veterinary administration due to the serious economic and epizootic threat to waterfowl production. Prophylactic treatment for DD includes attenuated live or inactivated vaccines. Moreover, the control of DD includes the monitoring of maternal derived antibody (MDA) levels in the offspring and antibody titers in the parent flock after vaccination. The aim of this study was to develop an ELISA for the detection of goose parvovirus (GPV) antibodies.

**Results:**

Two recombinant protein fragments derived from VP3 (viral protein 3) GPV, namely VP3ep6 and VP3ep4–6 with a mass of 20.9 and 32.3 kDa, respectively, were produced using an *Escherichia coli* expression system. These proteins were purified by one-step nickel-affinity chromatography, which yielded protein preparations with a purity above 95%. These recombinant proteins were useful in the detection of serum anti-GPV antibodies, and this was confirmed by Western blotting. However, recombinant VP3ep4–6 protein showed a greater ability to correctly identify sera from infected geese. In the next stage of the project, a pool of 166 goose sera samples, previously examined by a virus neutralization test (VN), was tested. For further studies, one recombinant protein (VP3ep4–6) was selected for optimization of the test conditions. After optimization, the newly developed ELISA was compared to other serological tests, and demonstrated high sensitivity and specificity.

**Conclusion:**

In conclusion, the VP3ep4–6 ELISA method described here can be used for the detection of antibodies to GPV in serum.

## Background

Goose parvovirus (GPV) infection, also known as Derzsy’s disease (DD), is a contagious, acute gastrointestinal disease that occurs in geese (*Anser anser*) and Muscovy ducks (*Cairina moschata*). The virus has a high mortality rate of up to 100% and causes a significant decrease in weight, loss of feathers on the back and neck and diarrhea [1]. It is particularly severe for young goslings and Muscovy ducklings with maternal antibody deficiency. The virus is transmitted horizontally and vertically. Egg shell contamination also occurs, resulting in hatchery contamination of disease free flocks [2, 3]. In Poland, the leader in goose production in Europe, the disease must be reported to the veterinary administration, due to the serious economic and epizootic threat to waterfowl production [4].

DD was first described by Fang in 1956 [5], and since then has been reported in many countries including England [6], Sweden [7], Poland [8], Hungary [9], Japan [10] and Taiwan [11]. Of the major goose farming countries, none are Derzsy’s disease-free. Despite widespread vaccination against GPV in the last 30 years, epidemiological data indicates its constant presence [12].

GPV belongs to the *Dependovirus* genus and *Parvoviridae* family. The virus has a small icosahedral capsid with a diameter of 20–22 nm assembled from 32 capsomeres [13, 14]. The genome consists of a 5106 nt single stranded DNA (ssDNA) encoding two main open reading frames (ORFs). The left ORF encodes the non-structural proteins, NS1 and NS2, which are involved in viral replication and regulation of capsid gene expression. The right ORF encodes three capsid proteins VP1 (viral protein 1) VP2 and VP3 [14–16]. All three structural proteins, VP1-VP3, are encoded by the same DNA sequence called a ‘basket structure’, and the entire amino acid sequence of VP2 and VP3 are contained within the carboxyl terminal portion of VP1 [16, 17]. The variability of the VP3 protein coding sequence is lower compared to the VP1 and VP2 structural proteins. The VP3 protein is the most abundantly expressed capsid protein of GPV [14, 18, 19], making it ideal for serological test applications.

For GPV prophylaxis, attenuated live or inactivated vaccines are administered. Nevertheless, the control of GPV still includes monitoring of maternal derived antibody (MDA) levels in the offspring and antibody titers in the parent flock after vaccination. Several serological methods have been employed for the detection of GPV antibodies including agar gel precipitation (AGP), virus neutralization assay (VN) [20], immunofluorescent assay (IFA) [21, 22], Western blotting [23] and ELISA [24–27]. IFA and AGP are low sensitivity methods that detect only a high level of antibodies and do not allow a quantitative determination. VN is an effective method, but it is time consuming and requires the propagation of the whole virus in cell culture, which is difficult because goose fibroblast tissue is unavailable for much of the year.

Enzyme-linked immunosorbent assay (ELISA) requires whole, purified virus particles or recombinant capsid proteins as a coating antigen. To date, only a few studies on the construction and use of recombinant GPV proteins have been published [18, 23, 27, 28]. The cost of an *Escherichia coli* (*E. coli)* expression system is low and enables easy purification of the protein produced. The strong advantage of *E. coli* over other expression systems, as well as the high immunogenicity of the VP3 protein were taken into account when designing this study.

In this paper, two immunodominant fragments of the VP3 protein of GPV (VP3ep6 and VP3ep4–6) were expressed and purified. An indirect ELISA was developed and compared with VN and another ELISA test. Finally, one of the purified proteins, VP3ep4–6, was used as the coating antigen in a new ELISA assay. It proved to be a sensitive, specific and ideal method for the detection of antibodies against GPV.

## Results

### Expression and purification of the GPV VP3 recombinant proteins

**Fig. 1:**
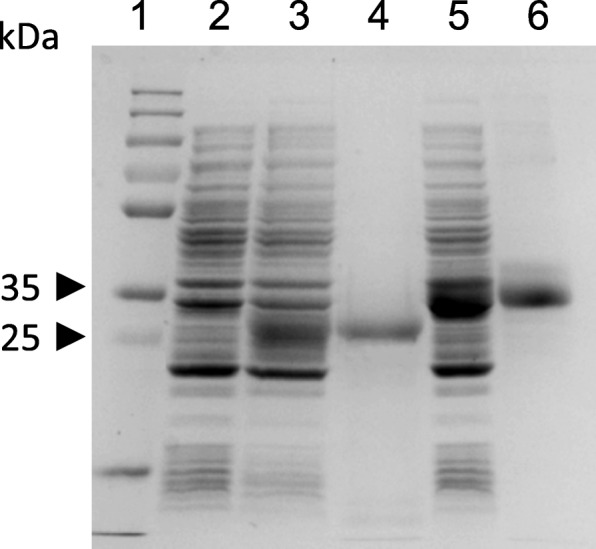
Expression and purification of VP3ep6 and VP3ep4–6 analyzed by SDS–PAGE. Lane 1 – molecular mass marker (ThermoFisher Scientific), lane 2 – soluble protein extracts from *E. coli* strain Rossetta (DE3) pLysS + pUET1, lane 3 – soluble protein extracts from *E. coli* strain Rossetta (DE3) pLysS + pUET1-VP3ep6, lane 4 – purified VP3ep6, lane 5 – soluble protein extracts from *E. coli* strain Rossetta (DE3) pLysS + pUET1-VP3ep4–6, lane 6 – purified VP3ep4–6

**Fig. 2:**
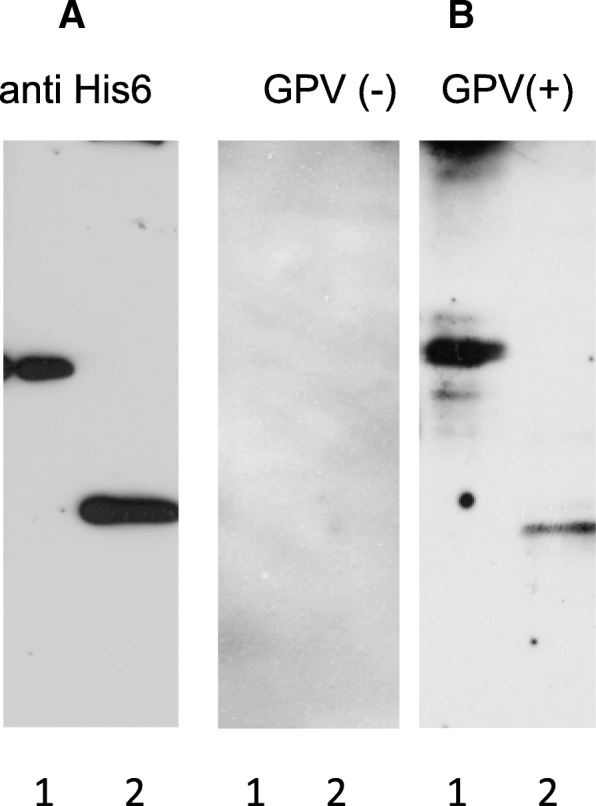
SDS-PAGE and Western blot analysis of recombinant antigens. Lane 1- VP3ep4–6, lane 2- VP3ep6. **a** Western blot with anti HIS6-tag antibody, **b** Western blot with negative and positive goose sera anti-GPV

Two GPV VP3 recombinant proteins (VP3ep4–6 amino acid 311–534 and VP3ep6 amino acid 418–534) containing six histidyl residues at N- and C-terminals were expressed as soluble proteins with a calculated molecular mass of 32.3 kDa and 20.9 kDa, respectively (Fig. 1). Purification of the GPV recombinant proteins was accomplished with a one-step chromatography procedure using metal-affinity chromatography with Ni^2+^ bound to iminodiacetic acid-agarose (Novagen) and resulted in a production yield of about 40 mg and 30 mg of purified proteins (VP3ep4–6 and VP3ep6, respectively) per liter of cell culture. The purification resulted in an electrophoretically homogeneous preparation with greater than 95% purity. Furthermore, Western blotting with specific HRP conjugated monoclonal mouse anti-His-tag antibodies confirmed the presence of recombinant proteins in the preparations (Fig. 2a).

### Reactivity of the recombinant proteins in Western blot

The immune-reactivity of the purified recombinant proteins (VP3ep4–6 and VP3ep6) was tested by Western blot analysis with two goose serum samples (one seropositive with specific IgG and the second without specific antibodies against GPV). IgG antibodies present in serum from the goose with DD, recognized both antigens, but immunoreactivity of the VP3ep4–6 protein was stronger. Furthermore, the immunoreactivity of these proteins with a serum sample from a healthy goose was not observed (Fig. 2b).

### Development of VP3-ELISA

The optimal dilution of the two antigens (VP3ep4–6 and VP3ep6), test sera and conjugate in the VP3 ELISA were determined using a checkerboard titration [27]. Of the 166 sera characterized using the VN test (72 negative and 94 positive), 30 negative and 30 positive sera were chosen for further VP3 ELISA optimization. The biggest differences in OD values for negative and positive sera were observed when using VP3ep4–6 as the coating protein at a final concentration of 5 μg/ml (OD_min_ 0.113, OD_max_ 0.914). The optimal dilution of the test sera was 1:50. Using this optimal dilution of coating VP3ep4 protein and sera, the optimal dilution of the HRP conjugated anti-goose IgG was found to be 1:500. The average OD value for 30 negative sera samples ranged from 0.068 to 0.340. The mean OD value in the ELISA was 0.150 (*n* = 30; min = 0.068; max = 0.340; SD = 0.0683). The cut-off was determined to be 0.355 (mean + 3SD; 0.150 + 3 × 0.068 = 0.355). The negative-positive threshold was consequently set at 0.355. Sera with an absorbance ≥0.355 were considered to be positive. Based on the above-mentioned criteria, all samples from the 30 negative control sera were negative (OD 0.068–0.340), and all 30 positive samples were positive (OD 0.360–0.846) in the new VP3-ELISA.

### Determination of repeatability and reproducibility

Twenty serum samples (10 positive sera and 10 negative sera) were selected at random and examined with both intra-assay and inter-assay variation, using a 24 h break in realization. The results showed that the intra-assay CV value (V = S_X_/X × 100%) of 20 samples ranged from 2.7 to 7.2%, and the inter-assay CV value ranged from 3.9 to 9.12%. The minor variation in results indicated that the new VP3-ELISA was reproducible.

### Comparison of the VP3ep4–6 ELISA and VN test

A total of 166 goose sera were examined in parallel comparing a VN test and the newly developed VP3-ELISA using VP3ep4–6 as the coating antigen. Using the VP3-ELISA, 90 goose serum samples were found to be positive (OD ≥ 0.355) and 76 were negative (OD < 0.355). In the VN tests, 94 samples were positive and 72 samples were negative (Table 1). In addition, 85 goose sera samples were determined to be positive in both cases, and 67 samples were judged as negative by both methods (Table 2). The total concordance rate (TC) between these two methods was 91.5% for the goose sera. Nine samples that were positive in the VN test were negative in the VP3-ELISA test, and five samples that were negative in the VN test were positive in the VP3-ELISA. The positive concordance rate (PC) of the VP3-ELISA in comparison with the VN test was 90.4%, whilst the negative concordance rate (NC) was 93%. Additionally, the specificity of the VP3-ELISA was calculated by the examination of goose sera that contained antibodies against Newcastle disease virus (NDV) and avian influenza virus (AIV). The OD value of applied NDV and AIV sera were ranged from 0.020 to 0.053. These results demonstrate that there was no cross-reactivity between VP3 protein of goose parvovirus (GPV) and other goose viruses.

**Table 1:** The titer of examined sera by different methods

Results	The amount of examined sera /ELISA titre	The amount of examined sera /VN titre
Positive	90 sera/≥0.355	94 sera/ SND_50_ ≥ 40
Negative	76 sera/≤0.355	72 sera/ SND_50_ ≤ 40
Total	166	166

**Table 2:** Results of 166 sera samples by VP3ep4–6 ELISA and VN test

VP3- ELISA	VN (goose sera)		
	Positive	Negative	Total
Positive	85	5	90
Negative	9	67	76
Total	94	72	166

Comparison of two ELISAs: the new VP3ep4–6 ELISA and an ELISA using whole GPV virion as the coating antigen.

Similar to the above-mentioned comparison, a total of 166 collected goose sera were examined in parallel using the VP3ep4–6 ELISA and the ELISA using the whole GPV virion as the coating antigen (Table 3). Receiver Operating Characteristics (ROC) with the Youden Index (0.361) [29] and Area Under Curve (AUC) were calculated. The sensitivity of the newly developed test was 97.8% (0.978) and the specificity was about 94% (0.94). AUC value was 92% (0.992). That results indicate high diagnostic value of the VP3-ELISA. Furthermore, the Kappa Cohen’s coefficient was calculated, and the result (0.829) indicates very good compatibility of both ELISA tests.

**Table 3:** Detection results of 166 sera samples by VP3ep4–6 ELISA and ELISA with the use of whole GPV virion as the coating antigen

VP3- ELISA	ELISA with the use of whole GPV virion as the coating antigen (goose sera)		
	Positive	Negative	Total
Positive	86	4	90
Negative	3	73	76
Total	89	77	166

## Discussion

Poland is the leader in goose production in Europe, and is fourth in goose production worldwide. The most infectious and acute viral disease among geese and ducks is DD. The aim of this study was to develop a simple and reliable method for the detection of GPV antibodies in order to analyze the immune status of vaccinated flocks or detect GPV infection. The conventional ELISA used in this study [unpublished data] and the ELISA reported by Kardi and Szegletes (1996) require propagation of GPV in cell culture goose embryo fibroblast’s (GEFs) and purification of virus particles before they can be used as the coating antigen. Most of the serological tests to detect GPV antibodies require cell culture, which is difficult due to goose fibroblast tissue unavailability for much of the year. IFA and AGP have low sensitivity. The VN test is considered to be very sensitive but is time consuming and requires at least a few days to assess the cytopathic effect. An ELISA based on recombinant protein as the coating antigen offers considerable advantages.

The huge advantage of *E. coli* over the other expression systems, as well as high immunogenicity of the chosen VP3 GPV protein, were taken into account in the design of this study. The studies conducted by Zhang (2010) revealed that among three viral protein (VP1-VP3) of GPV, VP3 has the highest expression level in *E. coli* cells. Ju and coauthors (2011), through the expression of GPV VPs in a Baculovirus Expression System (BES), have demonstrated immunogenicity and immunoreactivity of VP2 and VP3 proteins in geese. Moreover, other scientists also showed that the VP3 protein is highly immunogenic, easily inducing antibodies in both ducks and geese, and is produced abundantly during virus infection [28, 30]. Additionally, VP3 is the most stable protein of the GPV viral proteins, especially during the process of whole-virus antigen extraction. The high immunogenicity of the VP3 protein makes it applicable to goose immunization, which was confirmed by the latest results from Wang and co-authors [31], who describe the production of a bivalent goose vaccine with the use of the non-pathogenic strain, La Sota, of Newcastle disease virus (NDV) as a vector for VP3 GPV gene, resulting in successful immunization of birds against GPV and NDV.

After considering the above-mentioned information, two epitopes of GPV VP3 were selected. Both of the selected antigenic determinants VP3ep6 and VP3ep4–6 (with a mass of 20.9 kDa and 32.3 kDa, respectively) were characterized as having a high expression level in *E. coli* Rosetta (DE3) pLacI cells. This expression system and the method of purification by one-step metal affinity chromatography led to the production of milligram amounts of pure recombinant proteins (40 mg of VP3ep4–6 and 30 mg of VP3ep6) per liter of culture. The production efficiency of this system is much higher than the system previously reported by Zhang [25]. Furthermore, both recombinant antigens exhibited immunoreactivity in the presence of goose GPV positive sera using Western blot. In this work, the diagnostic value of two recombinant antigenic proteins as coating antigens in an ELISA assay was evaluated. A total 166 of goose sera samples, which were previously characterized by a VN test, were evaluated. Parameters such as concentration of the VP3ep4 and VP3ep4–6 antigens, serum dilution, conjugate dilution (conjugated anti-goose IgG conjugated with horseradish peroxidase, HRP), and the OD for positive and negative samples were optimized. The most significant difference in OD value for positive and negative sera was observed when using VP3ep4–6 antigen. The observed difference was almost nine-times higher (OD_min_ 0.113, OD_max_ 0.914), than when VP3ep6 was the coating antigen. Therefore, for further studies VP3ep4–6, the protein of higher molecular weight (32.3 kDa), was used.

The concordance of the VP3ep4–6 ELISA and the VN assay reached 91.5%, the sensitivity was 90.4%, and specificity was 93%. However, the comparison of the newly developed ELISA with the ELISA based on the whole virion of GPV as the antigen, showed very high sensitivity of 97.8% and a specificity of 94% based on ROC, AUC and Kappa Cohen’s coefficient. In this study, AUC reached 0.992, which indicates very good diagnostic value of the newly developed VP3-ELISA.

To determine the agreement of two tests, Zhang (2010) applied X^2^. In our research, a Kappa Cohen’s coefficient was used, which is a much better method for determining the compatibility of two measurements of the same variable under different conditions [32]. As a result, a score of 0.829 was obtained, which demonstrates very good compatibility of the two ELISA tests.

This VP3-ELISA is characterized by reproducibility and repeatability, which reached (V < 10%). The lack of a reaction when using sera containing antibodies against AIV and NDV also confirmed its specificity. In conclusion, the developed ELISA has many advantages over the conventional ELISA and the VN test such as its specificity, sensitivity, ease of implementation and low cost of protein production. These results demonstrate that it can be used as a better diagnostic tool in GPV infection.

## Conclusions

The ELISA developed in our study was compared to other serological tests and demonstrated high sensitivity and specificity. In conclusion, the VP3ep4–6 ELISA method can be used for the detection of antibodies to GPV in blood serum.

## Methods

### Serum samples

Serum samples (166) from geese and ducks suspected of DD from different commercial farms were collected. All the serum samples were checked with VN. Once samples were obtained from geese and ducks, the birds were directly utilized according to applicable in National Veterinary Research Institute procedures.

Goose sera positive for Newcastle disease virus (NDV) and avian influenza virus (AIV) antibodies were obtained from the Department of Poultry Disease in the National Veterinary Research Institute in Puławy, Poland. All the sera were inactivated for 30 min at 56 °C and stored at − 20 °C.

### Virus propagation

GPV strain 24/03 (Gen Bank accession number-GQ 468411) was isolated from the liver of geese aged 2.5 wks that had clinical signs of DD. The strain was propagated in goose embryo fibroblasts (GEF) prepared from 14 day-old embryos [according to Department of Poultry Disease procedure, National Veterinary Research Institute]. The cytopathic effect (CPE) was detected between 5 and 7 days post-infection. Cells were collected from the third virus passage. Viral particles were released from infected cells by three freeze-thaw cycles. DNA extraction was conducted according to the manufacturer’s instructions (QIAamp DNA Mini Kit, Qiagen, Hilden, Germany).

### Virus neutralization (VN)

The VN test was conducted in 96-well microtiter plates (Nuclon, Denmark) using 100 μl GEFs, containing 1 × 10^6^ cells/ml. Twofold serial dilutions of sera starting with 1:20 up to 1:10240, were mixed with an equal volume of Eagle’s (Sigma-Aldrich, America) 50 μl of GPV strain 24/03 were quickly added. The mixtures were incubated at 37 °C for 30 min. Each sample was tested three times. Evaluation was performed according to the Reed-Muench method [33]. Titers of 1.5 or greater were considered positive.

### Construction of recombinant plasmids

The nucleotide sequence of the GPV gene encoding two fragments of the VP3 protein (epitopes 4–6 and epitope 6) was obtained from the GenBank database (Accession U25749.1). The VP3 gene was assembled from synthetic oligonucleotides which were cloned into pMA-T using *Sfi*I and *Sfi*I clonic sites and was provided by Life Technologies (Thermo Fisher Scientific, USA). This construct was used as the template for the amplification of two fragments of the VP3 gene, using a standard PCR amplification protocol with the *Delta3* DNA polymerase (Blirt S.A., Poland). The VP3ep4–6 and VP3ep6 PCR products (a larger fragment encoding amino acids 509 to 732 and a shorter fragment encoding amino acids 616 to 732, respectively) were inserted into the pUET1 vector [34]. The first fragment of VP3ep4–6 DNA (corresponding to nucleotides 3963–4634) was obtained by PCR using the following primers: 5′-GTTCCGGATCCACAGTATCTCCTACAACC-3′ (forward) and 5′-CCCGGCGAAGCTTCAGATTTTGAGTTAG-3′ (reverse). The second fragment of VP3ep6 DNA (corresponding to nucleotides 4284–4634) was obtained by PCR using the following forward primer: 5′-CTAGTTCCGGATCCGGCAAAAATACCGAAG-3′ and the same reverse primer. The primers contained the *Bam*HI and *Hin*dIII recognition sequences (underlined) to facilitate cloning. The 697 bp and 379 bp PCR products were digested with both *Bam*HI and *Hin*dIII and inserted into the *Bam*HI and *Hin*dIII sites of pUET1. The resulting pUET1-VP3ep4–6 and pUET1-VP3ep6 constructs retained the open reading frame encoding amino acid residues 311 to 534 (epitopes 4–6) and 418 to 534 (epitope 6) of the VP3 protein and a cluster of six histidine residues for purification of the recombinant proteins by metal affinity chromatography at the N- and C-termini. The nucleotide sequences of the recombinant plasmids were verified using the dideoxy termination sequencing method.

### Expression and purification of the GPV VP3 recombinant proteins

The *E. coli* strain Rosetta (DE3) pLacI, transformed with pUET1-VP3ep4–6 and pUET1-VP3ep6 or non-recombinant pUET1 plasmid, was grown in LB media supplemented with 100 μg/ml of ampicillin and 34 μg/ml chloramphenicol at 30 °C overnight. Next, 1 l of LB medium, supplemented with the same antibiotics, was inoculated with 20 ml of the overnight culture. The cultures were grown at 30 °C with vigorous shaking until the optical density at 600 nm reached 0.4. Isopropyl-b-D-thiogalactopyranoside (IPTG) was added to a final concentration of 1 mM, and the cells were incubated with vigorous shaking for 18 h at the same temperature. The cells were harvested by centrifugation at 4000 *g* and the pellets were re-suspended in 30 ml of buffer A (20 mM Tris–HCl, pH 7.9, 500 mM NaCl, 5 mM imidazole, 0.1% Triton X-100). Next, the cells were disrupted by sonication and lysates were centrifuged at 9000 *g* at 4 °C for 20 min. The fusion proteins were purified from supernatants by metal-affinity chromatography using a Ni^2 + −^IDA-Sepharose column according to the manufacturer’s instructions (Novagen, USA). The GPV VP3 recombinant proteins were analyzed by SDS–PAGE on 12% acrylamide gels and stained with Coomassie blue. The concentration of purified proteins was determined by the Bradford method using bovine serum albumin (BSA) as a standard.

### Preparation of rabbit anti-goose IgG antibody

Goose IgG was isolated from normal goose serum by affinity chromatography on a Protein A-Sepharose column (ThermoFisher Scientific, US). After washing the column with PBS, the immunoglobulins were eluted with 0.1 M acetate buffer, pH 3.0. The fractions containing proteins were neutralized with 1 M Tris-HCl, pH 9.0 and dialyzed into PBS. The rabbits were immunized intramuscularly with a dose of 100 μg goose IgG in complete Freund’s adjuvant. Then, three times every 2 weeks, the same dose of immunogen in incomplete Freund’s adjuvant was delivered subcutaneously in the back of the neck. Two weeks after the last vaccination, the rabbits were euthanized by anesthesia with a mixture of ketamine and xylazine, followed by complete bleeding and the serum was separated after clotting. The rabbit antiserum (20 ml) was diluted with 20 ml of 0.15 M NaCl in 50 mM Tris-HCl, pH 7.5 and applied to a 5 ml immunoadsorbent column (10 mg goose IgG linked to Cyanogen bromide activated Sepharose 4B (BrCN-Sepharose 4B). After washing the column, the antibodies were eluted with 0.1 M Glycine-HCl buffer, pH 2.5. The fractions were neutralized with 1 M Tris-HCl, pH 9.0 and dialyzed into PBS.

### Labelling of rabbit antibodies against goose IgG with horseradish peroxidase

The conjugate was obtained by a modified periodate method. First, 4 mg HRP (Type VI, Sigma, USA) was dissolved in 1 ml of water and 200 μl of 0.1 M sodium periodate was added. The solution was incubated for 20 min at room temperature in a dark place and then dialyzed overnight into 2 mM acetate buffer, pH 4.5. Next, the solution was mixed with 6 mg of rabbit antibodies in 1 ml of 0.1 M carbonate buffer, pH 9.0 and incubated for 2 h at room temperature. Then 100 μl of sodium borohydride solution (5 mg/ml) was added and incubated for 1 h at room temperature. The conjugate was dialyzed against 0.15 M NaCl in 20 mM Tris-HCl buffer, pH 7.5 and stored with 0.02% merthiolate at 4 °C.

### Immunoblot analysis of recombinant antigens

The proteins were separated by SDS-PAGE using 12% resolving gels and transferred to PVDF membrane (0.45 μm pore size; Merck Millipore, Billerica, MA, USA). The membranes were blocked with 1% casein [0.1 M Tris-HCl, pH 8.0, 200 mM NaCl, 1% casein from bovine milk (Sigma-Aldrich)] overnight at 4 °C. Next, the membranes were washed with TBST (20 mM Tris, 150 mM NaCl, 0.1% Tween-20) and then incubated with anti-His-tag antibody (Sigma-Aldrich) or negative and positive goose antisera (anti-GPV) for 1 h at room temperature. After probing with an HRP conjugated secondary antibody for 1 h at room temperature, recombinant antigens were detected using SuperSignal West Pico Substrate (Thermo Fisher Scientific).

### ELISA with the whole GPV virion as the coating antigen

This optimized test is available in the Department of Poultry Disease in the National Veterinary Research Institute, Poland [license No. 183936 RP]. GPV was purified in GEF culture and is used as the coating antigen. An OD value greater than 0.200 indicates a positive sample, an OD value between 0.150 and 0.200 is a inconclusive result, whereas an OD value less than 0.150 is a negative result.

### VP3-ELISA procedure

Sera previously tested in the VN assay and described ELISA (10 positive and 10 negative), were used for optimization of the new ELISA. The plates were coated with two VP3 recombinant proteins: VP3ep4–6 and VP3ep6 in different concentration (5 μg/ml, 2.5 μg/ml, 1 μg/ml) in carbonate buffer (100 μl/well). The plates were incubated at 37 °C for 18 h and washed three times with phosphate-buffered saline (PBS) containing 0.05% Tween 20 (PBST). The antigen coated plates were blocked with BSA (0.2 mg BSA/100 μl/well, Sigma) for 1 h at 37 °C. After washing, goose sera diluted in blocking solution were added (1:50, 1:100, 1:200, 1:300, 1:400, 1:500; 100 μl/well) and plates were incubated for 1 h at 37 °C. After washing, the HRP-conjugated rabbit anti-goose IgG was diluted in blocking solution and added to the wells (1:100, 1:300, 1:500; 100 μl/well) and then incubated for 1 h at 37 °C. The plates were washed three times and incubated with substrate (ABTS, 100 μl/well, Sigma) for 30 min. The results were read in microplate reader at 405 nm (BIO-RAD, Japan).

### Statistical analysis

166 sera samples were used for comparing the results obtained from a new ELISA based on recombinant antigens and ELISA with the use of whole GPV preparation as the coating antigen. Specificity (NC), sensitivity (PC) and compatibility (TC) of adopted tests were performed. Receiver Operating Characteristic (ROC) with Youden’s Index, Area Under Curve (AUC) and Kappa Cohen’s coefficient were used (Statistica 10, Statistica Medical Set). The AUC value is a measure of goodness and relevance of a given model when less than 1 [32], while Kappa Cohen’s coefficient determined the agreement of two applied tests [35].

## Data Availability

The datasets used and analyzed during the study are available from the corresponding author on reasonable request.

## Abbreviations

AGP: Agar gel precipitation
AI: Avian influenza
AUC: Area under curve
BES: Baculovirus expression system
BSA: Bovine serum albumin
CPE: Cytopathic effect
DD: Derzsy’s disease
ELISA: Enzyme linked immunosorbent assay
GEF: Goose embryo fibroblasts
GPV: Goose parvovirus
IFA: Immunofluorescent assay
MDA: Maternal derived antibodies
NC: negative concordance rate
NDV: Newcastle disease virus
NS: Non structural
OD: Optical density
ORF: Open reading frames
PBS: Phosphate buffered saline
PC: positive concordance rate
ROC: Receiver operating characteristic
SPF: Specific pathogen free
TC: total concordance rate
VN: Virus neutralization test
